# Quantifying cervical cancer radiotherapy care gap: Baseline assessment prior to implementation of a digital Health App

**DOI:** 10.1016/j.tipsro.2026.100381

**Published:** 2026-02-04

**Authors:** Afua A. Yorke, Apollo Muramuzi, Lilie L. Lin, Kavuma Awusa, Solomon Kibudde, Peniel Tenkoramah Twum, Charles K. Yorke, Eric C. Ford

**Affiliations:** a University of Washington and Fred Hutchinson Cancer Center, Department of Radiation Oncology, Seattle, WA, United States; b The University of Texas, MD Anderson Cancer Center, Department of Radiotherapy, United States; c Uganda Cancer Institute, Division of Radiation Oncology, Uganda; d Global Communities, Accra, Ghana; e Nnalongo LLC, Everett, United States

**Keywords:** Digital health technologies, MHealth, Radiotherapy, Access to care, Patients Navigation, mHealth in Radiotherapy

## Abstract

**Purpose:**

Sub-Saharan Africa accounts for over one-third of global cervical cancer deaths, despite representing only 14% of the world’s female population. Radiotherapy (RT) services are essential in the treatment of cervical cancer, and the Uganda Cancer Institute (UCI) is the only oncology center with RT in a country of 50 million. Prior studies show most of the cervical cancer (CxCa) patients do not complete RT within the recommended timeframe of 6–8 weeks, and some miss their brachytherapy boost. This study analyzes care gaps to establish a baseline before implementing a digital health patient navigation app GLOCASSA.

**Methodology:**

We quantified radiotherapy care gaps among 104 cervical cancer patients (FIGO IB–IIIC) treated at the UCI between 2023 and 2024, representing 13% of the annual patient volume ethical approval was obtained. A mixed-methods approach was employed, combining retrospective review of radiotherapy charts with a custom mathematical framework for care gap assessment. Patient timelines were reconstructed from consultation through CT simulation, treatment initiation, completion, and follow-up, across three external beam radiotherapy regimens (45 Gy/15 fractions; 50 Gy/25 fractions; 50.4 Gy/28 fractions). We developed a Radiotherapy Care Gap (RCG) model incorporating professional benchmarks (ASTRO, ACR, ARS) to quantify delays, weighted by their clinical importance. Scores were normalized to reflect adherence (<1 expedited/incomplete; =1 standard; >1 delayed). Geospatial analyses were performed using ArcGIS Pro to measure travel distance from patient residence to UCI.

**Results:**

Among 104 cervical cancer patients treated with three EBRT regimens followed by brachytherapy, biologically equivalent doses were comparable across schedules (EQD2 ≈ 85–86 Gy). However, treatment completion within the recommended 6–8 weeks was rare, with on-time completion rates of 11% or lower at 6 weeks and ≤ 17% at 8 weeks. The majority of patients had Radiotherapy Care Gap Scores (RCGS) > 1, indicating significant delays, while only a small fraction achieved timely care (RCGS < 1). Extreme delays were observed, with some patients requiring over 30 weeks to complete therapy. Most patients (58%) were treated with 2D/3D techniques, though delays persisted across all modalities. Geospatial analysis showed a median travel time of 4 h to the radiotherapy center, and more than half of patients (53%) missed the recommended 6-week follow-up visit, highlighting substantial geographic and continuity-of-care barriers.

**Conclusion:**

Our findings suggest that patient navigation and follow-up support are major unmet needs. Digital health platforms that provide real-time reminders, enable remote symptom reporting, and facilitate coordination between patients and care teams offer promising solutions. Future directions include the implementation of the GLOCASSA-App as a targeted intervention to mitigate identified care gaps.

## Highlights

• Uganda Cancer Institute is sole RT center for 50M; major cervical RT completion gaps persist.
• Only 11% finished RT ≤6 weeks; some exceeded 30 weeks despite adequate EQD2.
• RCG model quantified delays vs ASTRO/ACR/ARS benchmarks across 104 patients.
• Median travel time 4 hrs; 53% missed the recommended 6-week follow-up.
• The GLOCASSA App addresses navigation gaps via reminders, symptom reporting, coordination.

## Introduction

Cervical cancer is the fourth most common cancer amongst women globally and the leading cancer type in Sub-Saharan Africa (SSA) [1]. Mortality rates are disproportionately higher in low-and-middle income countries (LMICs) compared to high-income countries, due, in part, to the limited availability of radiotherapy (RT) resources for treatment. Uganda is one country where there is a service gap with only one center with RT service the UCI in a country with a population of 50 million. For reference the International Atomic Energy Agency, recommends 1 radiotherapy machine per million people, Uganda has 3 radiotherapy linacs specifically Varian True Beams for the 50 million population [2]. Similarly, of the 51 countries in Sub-Saharan Africa, only 28 have radiotherapy facilities [3] with more than half of these centers (53%) located in South Africa. The concentration of radiotherapy services in urban areas significantly limits access for patients living in rural regions [4]. In Uganda, cervical cancer accounts for approximately 7,000 new cases annually and represents 20.5% [5]. However, only a subset of these patients are referred to and seen at the Uganda Cancer Institute radiotherapy unit, with locally advanced cervical cancer patients comprising approximately 27–30% of radiotherapy cases reported between 2021 and 2023 according to departmental data. A substantial proportion of cervical cancer patients likely never reach radiotherapy services, underscoring the importance of complementary interventions aimed at both access and continuity of care.

In a recent retrospective review of 196 cervical cancer patients (stages IB–IVA) conducted between November 2020-May 2021 at the UCI, only 11.7% adhered to the prescribed treatment plan completing all cycles of therapy (5 weeks of EBRT, 5 cycles of chemotherapy, and 3 brachytherapy insertions). The majority, 88.3%, did not complete the full course of prescribed chemoradiation [6].

Missed radiotherapy sessions significantly increase the risk of cancer recurrence and related mortality [7]. Cultural beliefs and practices can foster misconceptions and may promote mysticism about cancer, leading some patients to seek spiritual remedies instead of medical interventions [8]. Additionally, the stigma surrounding cancer-related illnesses, coupled with misperceptions of radiotherapy as “burning” or “electrocution” further hinder acceptance and adherence to treatment protocols.

Patient navigation in cancer care can potentially address this challenge. In the United States, a recent publication examined the state of patient navigation in cancer care a decade after its implementation [9], [10]. This initiative was developed to provide personalized support to patients, families, and caregivers, particularly those affected by health disparities by helping them navigate healthcare system barriers and ensuring timely access to quality care and psychosocial support throughout the cancer continuum, from diagnosis to survivorship [10], [11].

The world health organization (WHO) recognizes that digital health technologies could be used to maximize and improve the quality of life and patient safety in Africa implementing their mHealth program called “Be Happy Be Mobile” in 2018. Having an effective digital infrastructure in healthcare can give healthcare professionals a reliable way to coordinate the care of patients and improve communication with patients. A recent editorial [12] highlighted the benefits of digital health in Africa including better patient care to reduce the number of medical referrals, and cost of care on patients as well as the ease of access to the limited number of specialists given the lack of healthcare professionals particularly in the rural areas. In terms of infrastructure, mobile phone ownership has skyrocketed in SSA [13]. The Global Systems for Mobile Communications Association (GSMA) projected a surge in smartphone adoption by the end of 2023 in SSA, alongside Asia-Pacific and Latin America making mobile technology one of the many solutions to Africa’s healthcare problems. In SSA, mobile phone subscribers exceeded 482 million in 2022, with projections indicating a surge to over 690 million by 2030, marking a 50% increase in penetration. Currently, 51% of subscribers utilize smartphones, a figure expected to rise to 88% by 2030. The region boasts over 280 million mobile internet users, significantly contributing to digital transformation [14]. While 3G remains prevalent, 4G adoption is on the rise driven by younger consumers’ demand for faster speeds, with coverage projected to double to 45% [15]. CNN [16] reported in 2016 that more Africans have access to cell phones than clean water, echoing The Economist’s 2017 assertion of mobile phones’ pivotal role in the continent's economic development [17].With the widespread adoption of mobile phones and the internet as a communication tool, individuals with diverse medical conditions can access treatment plans, review their medical records, and consult with specialists at their convenience. Recent studies have demonstrated the effectiveness of mobile health interventions across various health domains in SSA [18], [19], [20], [21], [22]. Uganda’s rising adult literacy rate at 80.6% in 2022 from 70% in 2016 [23] provides a strong foundation for digital health adoption. Significantly, despite lower rates of individual phone ownership particularly in the rural areas where women are 22% less likely than men to own a device, most women report daily access to mobile phones through family or community sharing networks [24]. Because cervical cancer disproportionately affects women, this context presents a unique opportunity to implement digital health platforms to ensure that the women most at risk have an accessible, user-friendly platform designed to meet their needs, while leveraging existing patterns of shared phone use and rising digital literacy.

To be part of the solution, we developed the **Global Oncology Cancer Surveillance and Symptom Assessment mobile Application** (GLOCASSA-App) digital patient navigator to be used by patients during radiotherapy and post care surveillance. The purpose of the present study is to assess the access to care and gaps in care for patients with cervical cancer receiving treatment at UCI. This data will further inform our approach to implementation of the App.

## Methodology

We quantified the radiotherapy care gap among 104 cervical cancer patients, spanning early to advanced stages early to advanced stages (FIGO IB–IIIC) [25], [26] consistent with the late-stage disease presentation commonly low-resource settings [27]. Each one was scheduled to receive external beam radiotherapy and brachytherapy boost at the UCI (2023–2024). For context the UCI radiotherapy department treats over 800 new cervical cancer patients annually [28]. The study sample represent 13% of the patient population selected based on the availability of patient folders that had not yet been archived at the time of data collection. We employed a mixed-methods approach of retrospective clinical patient radiotherapy chart review and a custom mathematical framework for radiotherapy care gap assessment. We obtained approval from the Mulago Hospital Research and Ethics Committee.

a. Retrospective radiotherapy chart review

**Fig. 1:**
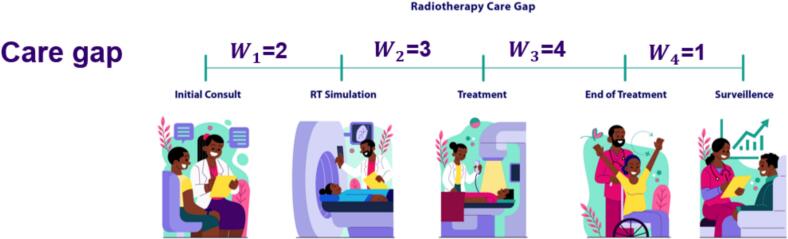
Illustration of the Radiotherapy Care Pathway highlighting key stages: Initial Consultation, RT Simulation, Treatment, End of Treatment, and Surveillance, with an emphasis on bridging care gaps. W = weight associated with the time interval.

Our work entailed a comprehensive review of patient medical records and was conducted to extract key timepoints along the radiotherapy care continuum. Data was collected for intervals from initial radiotherapy consultation to radiotherapy simulation, treatment start and end dates, and follow-up surveillance visits (**See** Fig. 1). This enabled the construction of individualized care timelines, allowing for the assessment of both timeliness and continuity of care delivery. Patients received one of three different external beam radiotherapy (EBRT) regimens, a. 45 Gy in 15 fractions (n = 37), b. 50 Gy in 25 fractions (n = 60), c. 50.4 Gy in 28 fractions (n = 7). Each followed by the same brachytherapy boost (8 Gy × 3 fractions). We analyzed the data for patients within each treatment group.

b. Development of a radiotherapy care gap quantification model

We developed a mathematical model described in **Equation 1** to quantify deviations from recommended standards of cervical cancer radiotherapy care. This model incorporates predefined clinical benchmarks. Although no globally uniform timelines exist for the radiotherapy care process, several professional bodies (ASTRO, ACR, ARS) [29]) and national cancer plans provide benchmarks. The recommended interval from consultation to CT simulation is approximately 1 week (5–7 working days), with the goal of simulating patients as soon as possible after consultation, particularly for curative intent. For urgent cases, a turnaround time of ≤ 48 h is recommended [29].The recommended interval from CT simulation to treatment planning including contouring, dosimetry, and quality assurance is 1–2 weeks: 2–5 working days for 3D CRT plans, and 7–10 working days for IMRT/VMAT or other complex plans. Overall, planning should be completed within 14 calendar days [30]. Following plan approval and physics QA, treatment initiation should occur within 2 days; for palliative cases, treatment can start the same or next day [31]. For cervical cancer treated with concurrent chemoradiotherapy and brachytherapy boost, the entire course measured from the first EBRT fraction to the last brachytherapy fraction should be completed within 56 days [32]. To reflect the clinical significance of each care interval, we assigned weighting factors to each care period based on physician input and team discussions. The intervals and their corresponding weights were as follows: initial consult to RT simulation (w_1_ = 2), RT simulation to treatment start (w_2_ = 3), treatment start to end-of-treatment (w_3_ = 4), and end-of-treatment to surveillance (w_4_ = 1). We prioritized the treatment-to-end-of-treatment interval (w_3_=4) the highest due to its critical role in treatment completion an essential factor in preventing recurrence. The RT simulation-to-treatment interval (w_2_=3) was next in priority, as delays during this phase can impact treatment effectiveness due to changes in patient body habitus or tumor size. For each patient, we computed the Radiotherapy Care Gap (RCG) and normalized it under the assumption that care was delivered exactly according to recommended standards. The final RCG score provided a quantitative measure of the magnitude of gaps relative to ideal standard care. By definition, a score of 1 indicated adherence to standard care, a score < 1 reflected expedited care or incomplete treatment, and a score > 1 represented delayed care. In addition to computing the care gap, we used the Spatial Join analysis too from ESRI ArcGIS Pro software [33] to compute geospatial information for both quantitative and qualitative analyses. Specifically, we calculated the distance between each patient's physical address listed in their patient charts and the nearest radiotherapy center in this case the UCI. In addition to computing the RCG score, we also computed the overall time it took patients in each in treatment group to complete their treatment within the recommended 6–8 weeks [34] to complete both EBRT and their brachytherapy boost.

## Results

**Fig. 2:**
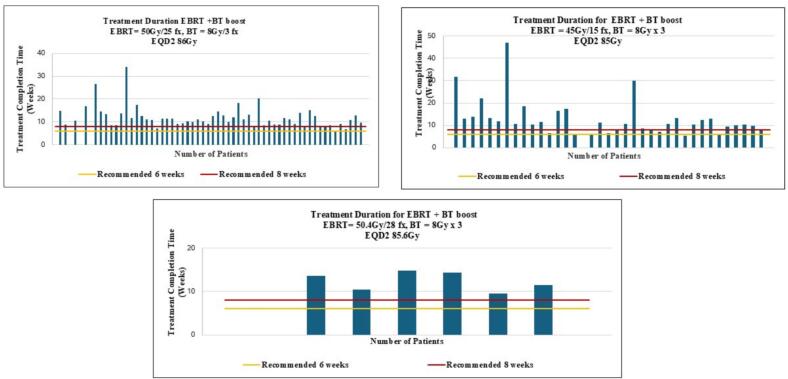
Treatment completion times (in weeks) for patients receiving EBRT and brachytherapy boost. The horizontal lines indicate the prescribed treatment durations of 6 weeks (yellow) and 8 weeks (red). (For interpretation of the references to colour in this figure legend, the reader is referred to the web version of this article.)

**Fig. 3:**
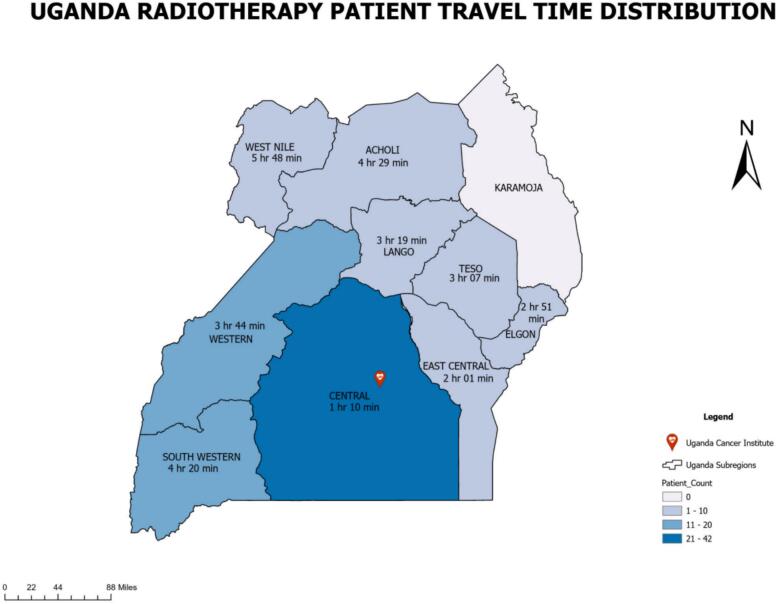
Spatial Distribution of Radiotherapy Patients in Uganda: Highlighting Accessibility and Regional Disparities with Average Travel Times to the Uganda Cancer Institute.

Among the 104 cervical cancer patients analyzed, three EBRT regimens were delivered, each followed by a brachytherapy boost of 8 Gy in three fractions: 45 Gy in 15 fractions (n = 37), 50 Gy in 25 fractions (n = 60), and 50.4 Gy in 28 fractions (n = 7). The corresponding EQD2 doses were 85 Gy, 86 Gy, and 85.6 Gy, respectively, indicating comparable dose intensity across regimens. Despite this, treatment completion within the recommended timeframe was uncommon. Only 11% of patients in the 45/15 group and 3% in the 50/25 group completed therapy within 6 weeks, while none in the 50.4/28 group did so. By 8 weeks, completion rates remained low at 8%, 17%, and 0%, respectively. Assessment of the Radiotherapy Care Gap Score (RCGS) a composite weighted measure of deviation from recommended timelines further highlighted substantial delays. Most patients fell into the RCGS > 1 category (75.7% for 45/15, 66.7% for 50/25, and 57.1% for 50.4/28), reflecting significant treatment interruptions. Only a small fraction achieved an RCGS < 1, consistent with timely care (5.4%, 8.3%, and 14.3%, respectively). Correlations between the RCGS and disease stage were weak and did not reach statistical significance. Among patients prescribed 45 Gy in 15 fractions**,** there was a moderate negative correlation (r = –0.32, p = 0.08), suggesting a trend toward lower RCGS scores in patients with more advanced disease, though this did not achieve significance. For patients receiving 50.4 Gy in 28 fractions (r = –0.07, p = 0.90) and 50 Gy in 25 fractions (r = –0.05, p = 0.73), correlations were negligible, indicating no meaningful relationship between disease stage and care gap within these regimens. suggesting that higher disease stage was marginally associated with lower RCGS scores. Table 1 provides a summary of these findings. Most patients (n = 59, 57.7%) were treated with 2D/3D techniques, while advanced modalities such as volumetric modulated arc therapy (VMAT) or intensity-modulated radiotherapy (IMRT) were used in 44 patients (42.3%). Fig. 2 illustrates the distribution of treatment completion times across the three EBRT regimens. Most patients exceeded the recommended treatment durations of 6 and 8 weeks, as indicated by the reference lines. In the 45 Gy/15 fraction cohort, several patients required more than 30 weeks to complete therapy, demonstrating extreme delays. Similarly, in the 50 Gy/25 fraction group, although a few patients completed treatment closer to the recommended timeframe, most still required more than 8 weeks. In the smaller 50.4 Gy/28 fraction cohort, none completed treatment within 8 weeks. These visual trends reinforce the pervasive delays observed across all regimens, aligning with the low on-time completion rates shown in Table 1. Data were incomplete for 13 patients, preventing determination of whether they returned for brachytherapy; these patients were excluded from the overall analyses. Geospatial analysis (Fig. 3) revealed a median travel time of approximately 4 h to the radiotherapy center, underscoring geographic access as a critical barrier to timely care. Additionally, 53% of patients did not return for the recommended post-treatment follow-up visit at 42 days (6 weeks), further highlighting challenges in continuity of care.

**Table 1:** External beam radiotherapy (EBRT) regimens, brachytherapy boost schedules, cumulative physical and biologically equivalent doses (EQD2), treatment completion rates at 6 and 8 weeks, and distribution of Radiotherapy Care Gap Scores (RCGS) among 104 cervical cancer patients.

EBRT fractionation (Daily)	45 Gy / 15 fx (n = 37)	50 Gy / 25 fx (n = 60)	50.4 Gy/25 (n = 7)
Brachytherapy Boost (weekly)	8 Gy /3 fx	8 Gy / 3 fx	8 Gy / 3 fx
Physical Dose	69 Gy	74 Gy	74.4 Gy
EQD2	85 Gy	86 Gy	85.6 Gy
% Of Treatment Completion at 6 weeks	11%	3%	0.0%
% Of Treatment Completion at 8 weeks	8%	17%	0.0%
% RCGS < 1	5.4%	8.3%	14.3%
% RCGS = 1	2.7%	0.0%	0.0%
% RCGS > 1	75.7%	66.7%	57.1%

## Discussion

This study provides a comprehensive baseline assessment of cervical cancer radiotherapy care at the UCI prior to the implementation of the GLOCASSA-App digital health app designed to enhance treatment completion and improve patient experience. Using a mixed-methods approach, we quantified care gaps across the radiotherapy continuum and developed an RCG scoring model that objectively characterizes deviations from established care timelines. Our findings highlight significant delays and variability in care delivery, underscoring critical barriers that impede optimal treatment outcomes in low-resource settings.

## Delays and inconsistencies in radiotherapy care delivery

Treatment delays in low-resource radiotherapy settings arise from a combination of system-level and patient-level factors. System-level contributors include limited radiotherapy machine availability, intermittent downtime related to maintenance or power instability, and high patient volumes that strain scheduling capacity. Patient-level barriers include long travel distances, reliance on public or informal transportation, limited availability of short-term accommodation near treatment centers, and financial constraints that affect treatment adherence. While digital health technologies cannot directly mitigate machine downtime, they can reduce secondary delays by improving appointment coordination, enabling early identification of missed or delayed fractions, facilitating rapid rescheduling, and supporting timely communication between patients and care teams. Digital symptom monitoring and patient navigation tools may also prevent treatment interruptions by identifying emerging toxicities or logistical challenges before they result in missed treatments. Together, these functions can improve continuity of care and reduce avoidable delays in radiotherapy delivery, even in settings with constrained machine availability.

Our analysis revealed pervasive delays in the delivery of definitive radiotherapy for cervical cancer, despite comparable prescribed dose intensities across fractionation regimens. Although patients received EBRT schedules ranging from 15 to 28 fractions, each followed by a brachytherapy boost, completion of treatment within the recommended 6–8-week window was rare. Fewer than one in five patients completed therapy on time, and in the 50.4 Gy/28 fraction cohort, none completed treatment within 8 weeks. These findings highlight the gap between prescribed intent and actual delivery in routine practice.

The Radiotherapy Care Gap Score (RCGS) further quantified these delays, with most patients across all regimens falling into the RCGS > 1 category, indicating significant deviations from standard timelines. Interestingly, the 15-fraction regimen intended as a hypo-fractionated approach to accelerate treatment in resource-limited settings had the highest proportion of patients with prolonged delays. This suggests that hypo-fractionation alone is insufficient to mitigate systemic barriers, and that structural issues, such as machine availability, patient flow, and care coordination, remain critical determinants of timeliness.

## Geospatial determinants of access

Geographic and continuity-of-care barriers further amplified these challenges. The median travel time of approximately 4 h to the radiotherapy center demonstrates the burden of geographic inaccessibility. Moreover, more than half of patients failed to return for the recommended 6-week post-treatment follow-up, reflecting persistent gaps in survivorship care and long-term monitoring. These findings emphasize that both timely treatment initiation and sustained engagement in care are compromised, with implications for disease control, toxicity management, and survival outcomes.

## Clinical utility of the radiotherapy care gap (RCG) score

The RCG Score provided a composite, quantitative measure of deviations from recommended treatment timelines. Across all regimens, the majority of patients had RCGS values > 1, reflecting substantial delays in care delivery. Importantly, this metric captured not only missed benchmarks at 6 or 8 weeks but also the degree of deviation from optimal schedules, offering greater granularity than binary on-time versus delayed measures. The ability of the RCG Score to stratify patients across treatment groups highlights its potential as a standardized tool for monitoring quality of care, identifying bottlenecks in delivery, and comparing performance across institutions. By quantifying timeliness in a reproducible way, the RCGS may serve as an important quality indicator in both research and clinical practice, especially in low-resource settings where delays are pervasive but under-measured.

## Implications for digital health intervention

The delays and inconsistencies identified in this study underscore the need for supportive interventions that go beyond optimizing radiotherapy dose and technique. Geographic barriers, reflected by a median travel time of four hours to the radiotherapy center, and high rates of missed post-treatment visits suggest that patient navigation and follow-up support are major unmet needs. Digital health platforms like GLOCASSA-App that provide real-time reminders, enable remote symptom reporting, and facilitate coordination between patients and care teams offer promising solutions. By integrating adherence tracking with care navigation, digital tools could reduce missed appointments, flag patients at risk of treatment interruption, and improve communication between providers and patients. In this way, digital health interventions could complement system-level improvements, bridging the gap between prescribed care and delivered outcomes.

## Study limitations and future direction

This study has several limitations. Its retrospective design and reliance on clinical records may not fully capture contextual factors or patient-reported experiences that contributed to treatment delays. Chemotherapy data were also unavailable and therefore not accounted for, which limits the ability to evaluate the impact of concurrent treatment on overall care timelines. In addition, while the RCG model incorporates a weighted scoring system, further validation is required across multiple institutions and cancer types to ensure its generalizability. Looking ahead, our ongoing work is focused on the implementation and evaluation of the GLOCASSA App, with particular attention to its potential to improve RCG scores, enhance patient-reported outcomes, and strengthen adherence and continuity of care.

Additionally, it should be noted that Formal evaluation of acceptability, usability, and feasibility was beyond the scope of this study but represents the next phase of this work. Our implementation approach includes engagement with clinicians, nurses, and patient navigators to assess workflow integration, as well as patient-centered usability testing through qualitative interviews and co-design workshops. Currently similar methods are being used in a parallel study at a comparable institution to inform app refinement.

## Funding Statement

AAPM International Council Global Health Seed Funding.

## Declaration of competing interest

The authors declare that they have no known competing financial interests or personal relationships that could have appeared to influence the work reported in this paper.
